# Supplementary material for: Intrinsic Lipid Curvature and Bilayer Elasticity as Regulators of Channel Function: A Comparative Single-Molecule Study
Source: Int J Mol Sci. 2024 Feb 27;25(5):2758. doi: 10.3390/ijms25052758 (PMC10931550; doi:10.3390/ijms25052758)
Supplement: Supplementary file 1 [file ijms-25-02758-s001.zip › ijms-2836278-supplementary.pdf]

Md. Ashrafuzzaman, Roger E. Koeppe II, and O. S. Andersen

Intrinsic lipid curvature and bilayer elasticity as modulators of channel function: a comparative single-molecule study

Supplementary Information

Ijms-2836278-supplementary.docx
Draft of February 27, 2024

SUPPLEMENTARY RESULTS: ***Lipid head group effects on Alm and gA channel function***

The amphiphile-induced changes in Alm channel function, with amphiphiles that promote negative and positive curvatures having similar effects, are surprising given the previously established relation between maneuvers that alter lipid intrinsic curvature and the probability of being in the higher conductance states [1, 2]. We therefore examined whether the choice of *n*-decane—rather than *n*-hexadecane, which was used by Keller et al. and Bezrukov et al.—could account for the difference. It did not (Fig. S1).

Effect of DOPE

We first explored the effect of adding dioleoylphosphatidylethanolamine (DOPE) to the bilayer forming solution, which causes negative changes in curvature [1].

Figure S1 near here

Single-channel current traces obtained with Alm in DOPE bilayers (Fig. S1top) are similar to those obtained in DOPC in the presence of amphiphiles, except that the probability of the channels residing in the higher conductance levels increases as the mole-fraction of DOPE is increased (Fig. S1bottom). The change in the probability of residing in level 2 relative to level 1 increases almost 10-fold when the DOPE mole-fraction is increased from 0 to 1; the corresponding increase in the probability of residing in level 3 relative to level 1 is about 100-fold. These results are in near-quantitative agreement with those of [1]. indicating that the choice of hydrocarbon solvent is of little consequence.

The current levels did not vary as the lipid composition of the host bilayer was varied from DOPC to DOPE (Table S1),

----
Table S1 near here
----

indicating that the change in head group structure has little impact on the channel structure. Surprisingly, given the shift toward higher current levels in DOPE membranes, we needed to add 10-fold more Alm in the DOPE experiments. as compared to the DOPC experiments, in order to have comparable channel activities. Similar results were observed by [1].

Titrating DOPS

We also examined how Alm channel activity in dioleoylphosphatidylserine (DOPS) bilayers is modulated by changes in intrinsic lipid curvature caused by titrating the phospholipid head groups. DOPS phase preference varies as a function of pH [3] and the intrinsic curvature of DOPS becomes more negative as the pH is decreased to ~2 [4]. As would be expected from the results of Keller et al. [1] and the above results with DOPE, the probability of ALM to reside in different current levels varies as a function of pH [2, 5], with the higher current levels being stabilized as the pH is decreased. The results of Bezrukov and colleagues were obtained in *n*-hexadecane-containing bilayers, and we examined how Alm function varies as a function of pH in DOPS/*n*-decane bilayers. Fig. S2 (top and middle) shows current traces obtained in 0.1 M NaCl at pH 7 and 4. There is little effect of pH on the amplitude of the different current levels (Table S1), indicating that the changes in pH have little effect on channel structure.

Figure S2 near here

At pH 4, where the DOPS intrinsic curvature is more negative, there is an increased probability of observing the higher current levels (Fig. S2bottom). These pH-dependent changes are, in part, due to bilayer-mediated effects (changes in intrinsic lipid curvature, as the pH changes do not alter the ratio of $gA^{-}(13)$ and AgA(15) single-channel lifetimes [6]). But decreasing the pH is likely to have additional effects because there is a pH-dependent change in W_2/W_1 (and W_3/W_1) also in DOPC bilayers (Fig. S3), suggesting that Alm channels may have an intrinsic pH-dependence.

Figure S3 near here

To get insight into the pH-dependent changes in Alm channel function in DOPC bilayers, we examined the effect of changes in pH on gA$^-$(13) and AgA(15) channels in these membranes. At pH 7.0: τ_{13} = 13.8 ± 1.7 ms and τ_{15} = 163 ± 46 ms. At pH 4: τ_{13} = 11.6 ± 1.9 ms, $\tau_{AgA(15)}$ = 161 ± 10 ms. The lifetime ratios, τ_{15}/τ_{13}, differ minimally, being 11.8 ± 2.8 at pH 7.0 and 14.1 ± 2.1 at pH 4.0 (three independent experiments at each pH). The pH-dependent changes in Alm channel function are unlikely to result from altered bilayer properties; they more likely result from direct pH-dependent changes in Alm channel function. It is in this context noteworthy that there is a titratable glutamate close to the carboxyterminus in Alm [7], see also Fig. 1 in the main text.

Surprisingly, however, the current level amplitudes are less in DOPS vs. DOPC (or DOPE) bilayers (Table S1), which raises the question whether the phosphatidylserine head groups have direct effect on Alm channel structure—in addition to bilayer-mediated effects. We did not explore this question.

To ascertain whether increasing the mole-fraction of DOPE in DOPC:DOPE membranes alter bilayer elasticity, we did experiments with gA$^-$(13) and AgA(15) in DOPC, DOPC:DOPE (1:1) and DOPE bilayers, see also [6]. Adding DOPE to DOPC reduces the lifetimes of both with gA$^-$(13) and AgA(15) channels (Fig. S4), with similar relative changes in the lifetimes of the gA$^-$(13) and AgA(15) channels. $\tau_{15}^{\mathrm{DOPE}} / \tau_{15}^{\mathrm{DOPC}}$ is about three-fold less than the changes reported by Rostovtseva et al. [8] for "solvent-free" membranes. With reference to Eq. 15 in the main text: $\tau_{15}^{\mathrm{DOPC}} \cdot \tau_{13}^{\mathrm{PC:PE}} / (\tau_{15}^{\mathrm{PC:PE}} \cdot \tau_{13}^{\mathrm{DOPC}}) = 1.04 \pm 0.35$ and $\tau_{15}^{\mathrm{DOPC}} \cdot \tau_{13}^{\mathrm{DOPE}} / (\tau_{15}^{\mathrm{DOPE}} \cdot \tau_{13}^{\mathrm{DOPC}}) = 1.18 \pm 0.28$, which differ at $p < 0.05$.

Figure S4 near here

This relative invariance in the ratio of the single-channel lifetimes shows that the changes in head group composition (replacing DOPC by DOPE) produce only modest changes in bilayer elasticity, with DOPE membranes being stiffer than DOPC and DOPC:DOPE (1:1) membranes by 4 ± 4 kJ/(mol·nm^2). We conclude that replacing DOPC by DOPE in *n*-decane-containing bilayers alters primarily the lipid intrinsic curvature, see also [6].

Supplementary Table S1

Alm channel current levels in bilayers with different head groups

	Level 0 (pA)	Level 1 (pA)	Level 2 (pA)	Level 3 (pA)
DOPC	4.5 ± 0.2	18.0 ± 0.5	38.4 ± 0.8	61 ± 1
DOPC pH 4	4.4 ± 0.3	17.1 ± 1.2	36.5 ± 2.1	59 ± 2
DOPC:DOPE (1:1)	4.5 ± 0.1	18.2 ± 0.3	39.8 ± 0.3	63 ± 1
DOPE	4.4 ± 0.3	17.6 ± 0.6	38.3 ± 0.7	61 ± 1
DOPS	3.5 ± 0.6	11.1 ± 0.9	25.5 ± 2.3	44 ± 3
DOPS 0.1 M NaCl, pH 7.0	2.1 ± 0.3	6.3 ± 1.5	9.8 ± 1.1	14.0 ± 0.6
DOPS 0.1 M NaCl, pH 4.0	3.0 ± 0.3	7.1 ± 0.5	11.0 ± 0.5	14.7 ± 0.6

Mean ± SD (based on three or more independent experiments)

Except where noted, the electrolyte was 1.0 M NaCl, pH 7.0; 150 mV.

Supplementary Figure S1:

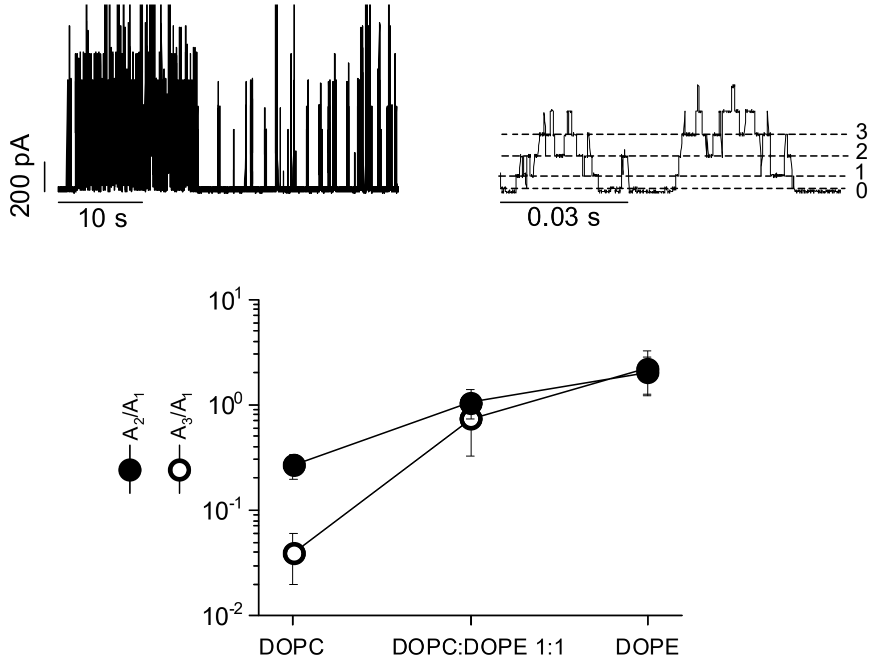

Figure S1: Alm channel function in DOPE (and DOPC:DOPE 1:1) bilayers formed using *n*-decane. Top: Alm current trace; the trace segment to the right is shown at an expanded timescale. The current levels (0, 1, 2, 3, etc.) are indicated in the trace. Bottom: Changes in the distribution of time Alm channels reside in current levels 2 and 3, relative to level 1, as function of the mole-fraction of DOPE. 1.0 M NaCl, pH 7.0, 150 mV. Mean ± SD, based on three independent experiments at each bilayer composition.

Supplementary Figure S2:

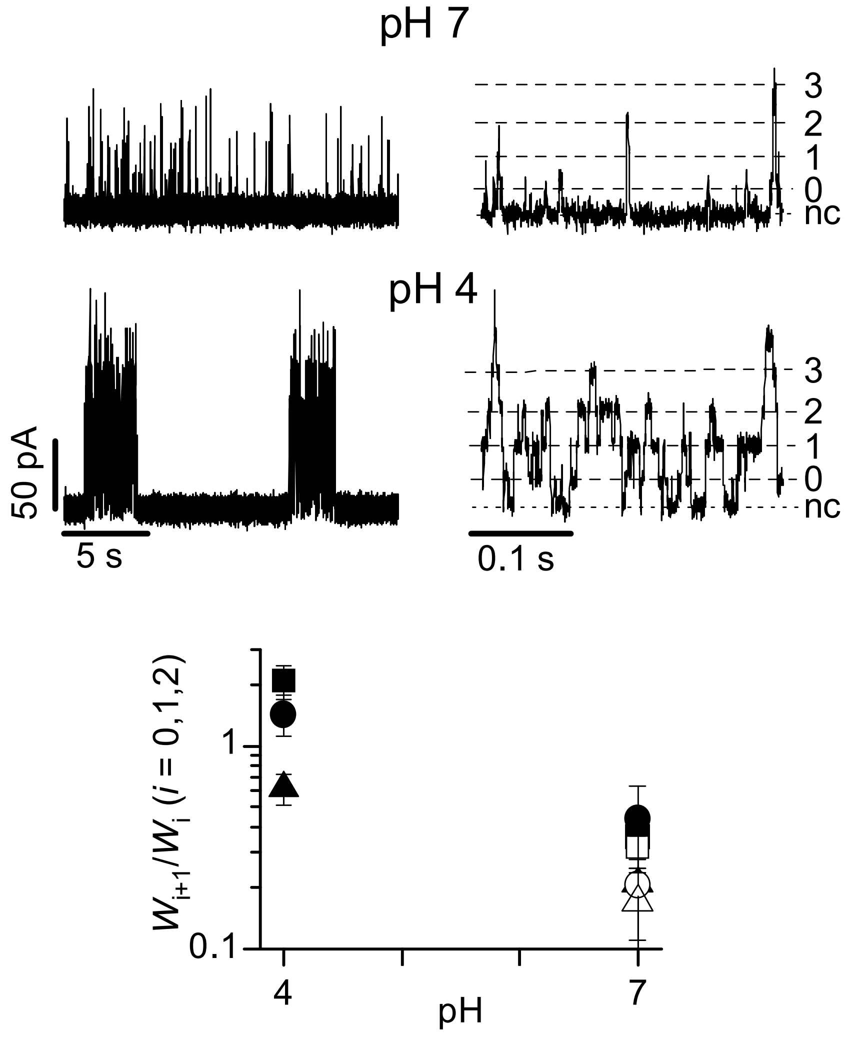

Figure S2: Alm channels in DOPS/*n*-decane at pH 7.0 and 4.0. Top and middle: 20 s current traces using 0.1 M NaCl; the trace segments to the right are shown at an expanded timescale at pH 7 (top) and 4 (middle); the vertical calibration at the left applies to both panels. The current levels (0, 1, 2, 3, etc.) are indicated in the expanded trace segments. Lower panel: Relative probabilities (W_{i+1}/W_i, $i = 0, 1, 2$), cf. Eq. 2 in the main text, of observing the higher Alm channel current levels in 0.1 M and 1.0 M NaCl solutions. W_1/W_0: ■ and □. W_2/W_1: ● and ○. W_3/W_2: ▲

and Δ. Filled symbols, 0.1 M NaCl; open symbols, 1.0 M NaCl. 150 mV. Based on three independent experiments at each experimental condition, each with one to three measurements.

Supplementary Figure S3:

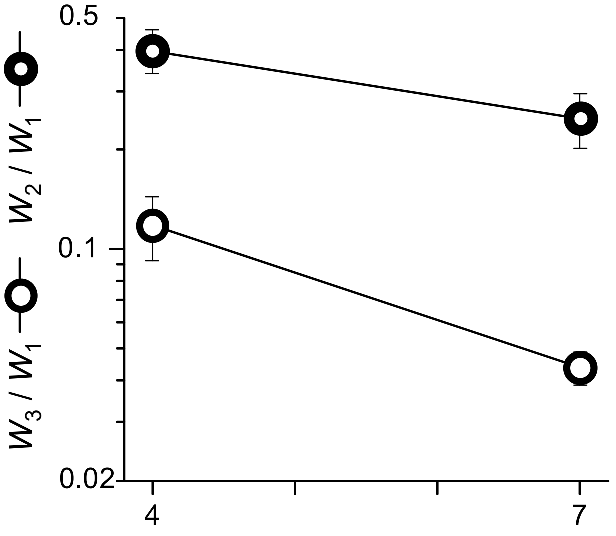

Figure S3: pH dependence of the ratio of the probability of observing the higher current levels in Alm channels relative to level 1 ($W_i/W_1 = A_i/A_1$; $i = 2, 3$), cf. Eq. 2 in the main text. DOPC, 1.0 M NaCl, 150 mV. Three independent experiments at each pH, each with one to three measurements. $(W_2/W_1)_{\text{pH 4}}/(W_2/W_1)_{\text{pH 7}} = 1.7 \pm 0.4$, $(W_3/W_1)_{\text{pH 4}}/(W_3/W_1)_{\text{pH 7}} = 2.8 \pm 0.7$ (Mean ± SD).

Supplementary Figure S4:

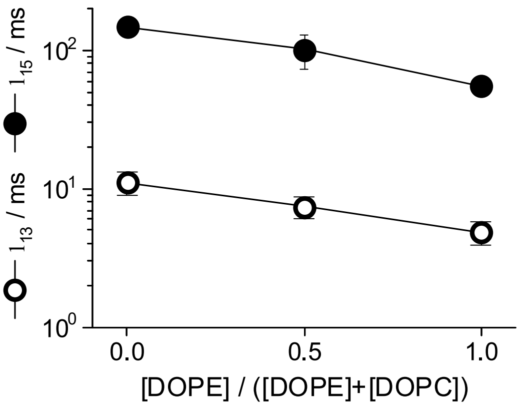

Figure S4: gA channel function in DOPC, DOPE (and DOPC:DOPE 1:1) bilayers formed using *n*-decane. Changes in the lifetimes of AgA(15) and $gA^{-}(13)$ channels as function of the mole-fraction of DOPE. 1.0 M, pH 7.0, 200 mV. $\tau_{15}^{DOPE} / \tau_{15}^{DOPC} = 0.37 \pm 0.06$; $\tau_{13}^{DOPC} / \tau_{15}^{DOPC} = 0.074 \pm 0.009$; $\tau_{13}^{PC:PE} / \tau_{15}^{PC:PE} = 0.075 \pm 0.008$; and $\tau_{13}^{DOPE} / \tau_{15}^{DOPE} = 0.087 \pm 0.005$, where the superscript "PC:PE" denotes the DOPE:DOPC = 1:1. Using the Bonferroni correction for multiple comparisons, the three ratios do not differ ($p > 0.2$). Calculating the changes in τ_{13} / τ_{15} for the three membranes: $\tau_{15}^{DOPC} \cdot \tau_{13}^{PC:PE} / (\tau_{15}^{PC:PE} \cdot \tau_{13}^{DOPC}) = 1.04 \pm 0.35$ and $\tau_{15}^{DOPC} \cdot \tau_{13}^{DOPE} / (\tau_{15}^{DOPE} \cdot \tau_{13}^{DOPC}) = 1.18 \pm 0.28$, which differ at $p < 0.05$. Based on three independent experiments at each bilayer composition.

1. Keller, S. L.; Bezrukov, S. M.; Gruner, S. M.; Tate, M. W.; Vodyanoy, I.; Parsegian, V. A., Probability of alamethicin conductance states varies with nonlamellar tendency of bilayer phospholipids. *Biophys. J.* **1993,** 65, (1), 23-27.
2. Bezrukov, S. M.; Rand, R. P.; Vodyanoy, I.; Parsegian, V. A., Lipid packing stress and polypeptide aggregation: alamethicin channel probed by proton titration of lipid charge. *Faraday Discuss.* **1998,** 111, 173-83; discussion 225-46.
3. de Kroon, A. I. P. M.; Timmermans, J. W.; Killian, J. A.; de Kruijff, B., The pH dependence of headgroup and acyl chain structure and dynamics of phosphatidylserine, studied by ^{2}H-NMR. *Chem. Phys. Lipids* **1990,** 54, (1), 33-42.
4. Fuller, N.; Benatti, C. R.; Rand, R. P., Curvature and bending constants for phosphatidylserine-containing membranes. *Biophys. J.* **2003,** 85, (3), 1667-1674.
5. Aguilella, V. M.; Bezrukov, S. M., Alamethicin channel conductance modified by lipid charge. *Eur. Biophys. J.* **2001,** 30, (4), 233-41.
6. Maer, A. M.; Rusinova, R.; Providence, L. L.; Ingólfsson, H. I.; Collingwood, S. A.; Lundbæk, J. A.; Andersen, O. S., Regulation of Gramicidin Channel Function Solely by Changes in Lipid Intrinsic Curvature. *Frontiers in Physiology* **2022,** 13.
7. Rinehart, K. L. J.; Cook, J. C. J.; Meng, H.; Olson, K. L.; Pandey, R. C., Mass spectrometric determination of molecular formulas for membrane-modifying antibiotics. *Nature* **1977,** 269, (5631), 832-833.
8. Rostovtseva, T. K.; Petrache, H. I.; Kazemi, N.; Hassanzadeh, E.; Bezrukov, S. M., Interfacial polar interactions affect gramicidin channel kinetics. *Biophys. J.* **2008,** 94, (4), L23-L25.
